# PP61 A Decade Of Enhanced Patient And Citizen Involvement In Health Technology Appraisal In Scotland

**DOI:** 10.1017/S0266462324002320

**Published:** 2025-01-07

**Authors:** Jennifer Dickson, Daniel Cairns, Jackie McCormack, Kate Russell

## Abstract

**Introduction:**

Over the past 10 years, the Scottish Medicines Consortium (SMC) has strived to strengthen the involvement of patients and citizens in its work and improve public understanding of health technology appraisal (HTA). A series of innovations have been brought in since 2014 to increase levels of engagement and satisfaction of participants. This work describes these and their impact.

**Methods:**

SMC has introduced numerous transformational changes since 2014. Innovations include a new Patient Group Partner (PGP) registration system, strengthened patient group submission process, participation of patient representatives at Patient and Clinician Engagement (PACE) and SMC Committee meetings, embargoed early release of SMC decisions to PGPs, provision of Summary Information for PGPs (from submitting company), and revised role of public representatives. The creation of the SMC Public Involvement Network Advisory Group has underpinned these achievements, as has the provision of comprehensive one-to-one support, information and education to PGPs. SMC has continually evaluated the satisfaction of participating PGPs using an online questionnaire.

**Results:**

There has been a sustained increase over the past 10 years in both the number of patient groups engaging with SMC and the number of PGP submissions that SMC receives. Adopting a continuous improvement approach, working in partnership with PGPs and public representatives, has helped SMC to ensure that stakeholders in the HTA process are effectively engaged and informed about the HTA of new medicines in Scotland. Surveys of public involvement in SMC consistently show an extremely high level of satisfaction from PGPs who work with SMC, with most PGPs consistently rating their experience of working with SMC as excellent.

**Conclusions:**

Over the past 10 years, SMC has strived to strengthen how it involves citizens and patient representatives in HTA. Various innovations and a continuous improvement approach have helped to ensure that there are high levels of satisfaction and understanding of the HTA process from patient groups who engage with SMC. This is underpinned by a partnership approach to working.

